# Giant bladder stone presenting with renal failure 15 years after ileocystoplasty augmentation: A case report

**DOI:** 10.1016/j.eucr.2026.103594

**Published:** 2026-09-04

**Authors:** Amir Alinejad Khorram

**Affiliations:** Urology Department, Shohada-e-Tajrish Hospital, Shahid Beheshti University of Medical Sciences, Tehran, Iran

## Abstract

A 68-year-old man with a history of ileocystoplasty performed 15 years prior presented with weakness and lethargy but no urinary symptoms. Despite the absence of typical complaints, imaging revealed a 10 × 9.8 cm giant bladder stone causing severe bilateral hydroureteronephrosis and renal failure. Following emergency dialysis, bilateral nephrostomy placement, and cystolithotomy, renal function recovered. This case highlights a giant stone within a bowel-segment urinary reservoir that caused silent obstruction and life-threatening renal failure, underscoring the need for lifelong follow-up after cystoplasty.

## 1 Introduction

Urinary stones are among the most common reasons for referral to urology clinics. Bladder stones account for approximately 5% of all urinary stone cases and are often associated with predisposing factors such as chronic bacteriuria, voiding dysfunction, or, in some cases, the presence of an intravesical foreign body 1, 2, 3. Stones weighing more than 100 g and exceeding 4 cm in diameter are considered "giant bladder stones" and are rare.^1^

Bowel-segment urinary reconstruction may increase the risk of urinary stone formation. The risk of stone formation has been shown to be lower in ileal neobladders compared to other methods; nevertheless, planned follow-up for prevention is recommended for all patients following enterocystoplasty and neobladder reconstruction.^4^

In this article, we report a unique case of a patient who developed renal failure 15 years after ileocystoplasty augmentation surgery due to a giant bladder stone.

## 2 Case report

A 68-year-old man presented to the emergency department with complaints of weakness and lethargy. His history of opium and alcohol addiction, along with low socioeconomic status, had resulted in poor healthcare engagement. During history-taking, he reported having undergone ileocystoplasty augmentation surgery approximately 15 years earlier. His surgical history was also notable for emergency laparotomy for a perforated peptic ulcer 8 years prior, and a failed incisional hernia repair 3 years prior. In the 15 years following bladder augmentation, bladder emptying was performed regularly every 8 hours using a 10-Fr Nelaton catheter (clean intermittent catheterization). Due to a lack of proper follow-up, the patient did not undergo any surveillance cystoscopy or metabolic evaluation. In recent years, he had experienced intermittent episodes of partial bowel obstruction, which had not been properly managed.

**Fig. 1:**
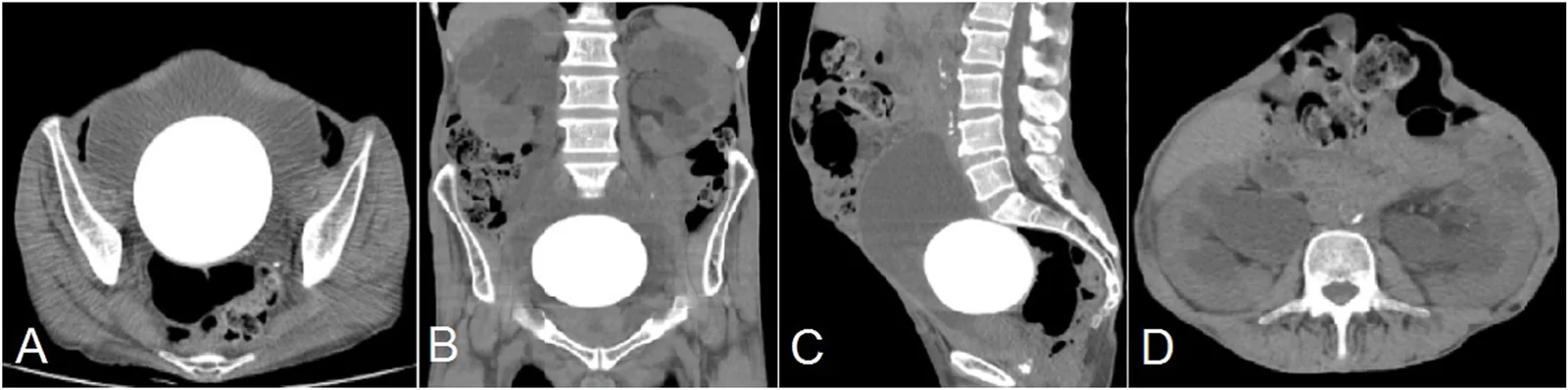
Computed tomography scan. Axial (A), coronal (B), and sagittal (C) views of the bladder, and axial view of the kidneys (D), demonstrating a large bladder stone and severe bilateral hydroureteronephrosis.

**Fig. 2:**
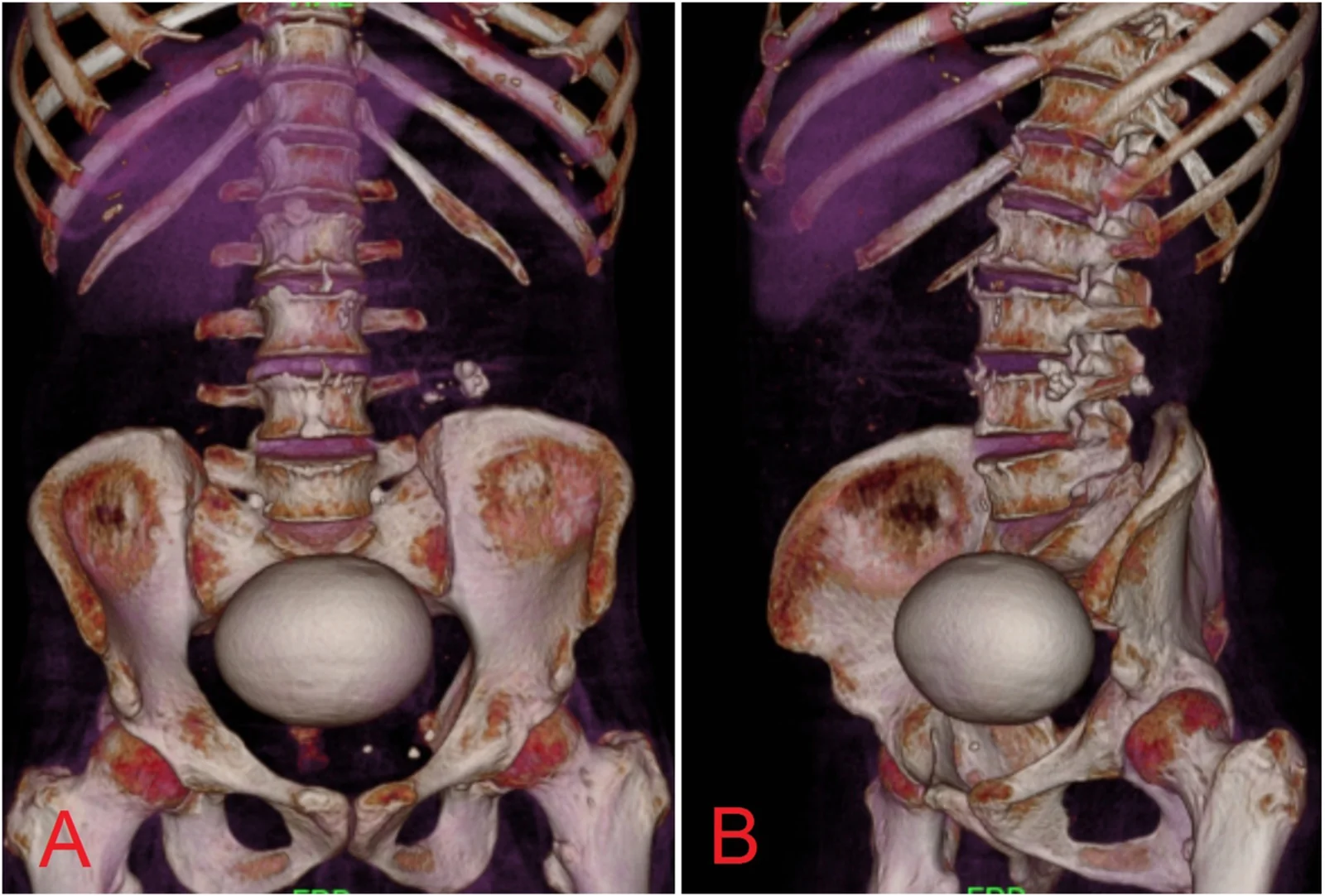
Three-dimensional computed tomography reconstruction illustrating a giant bladder stone, anterior (A) and lateral (B) views.

Clinical examination revealed a large incisional hernia in the abdominal midline and a stony, suprapubic mass on abdominal palpation. Laboratory findings indicated renal dysfunction (creatinine: 9.4 mg/dL, potassium: 6.1 mEq/L, pH: 7.25). Urine culture additionally showed infection with *Klebsiella pneumoniae*. Computed tomography (CT) revealed a giant bladder stone measuring 10 × 9.8 cm in diameter, with severe bilateral hydroureteronephrosis (Fig. 1, Fig. 2).

**Fig. 3:**
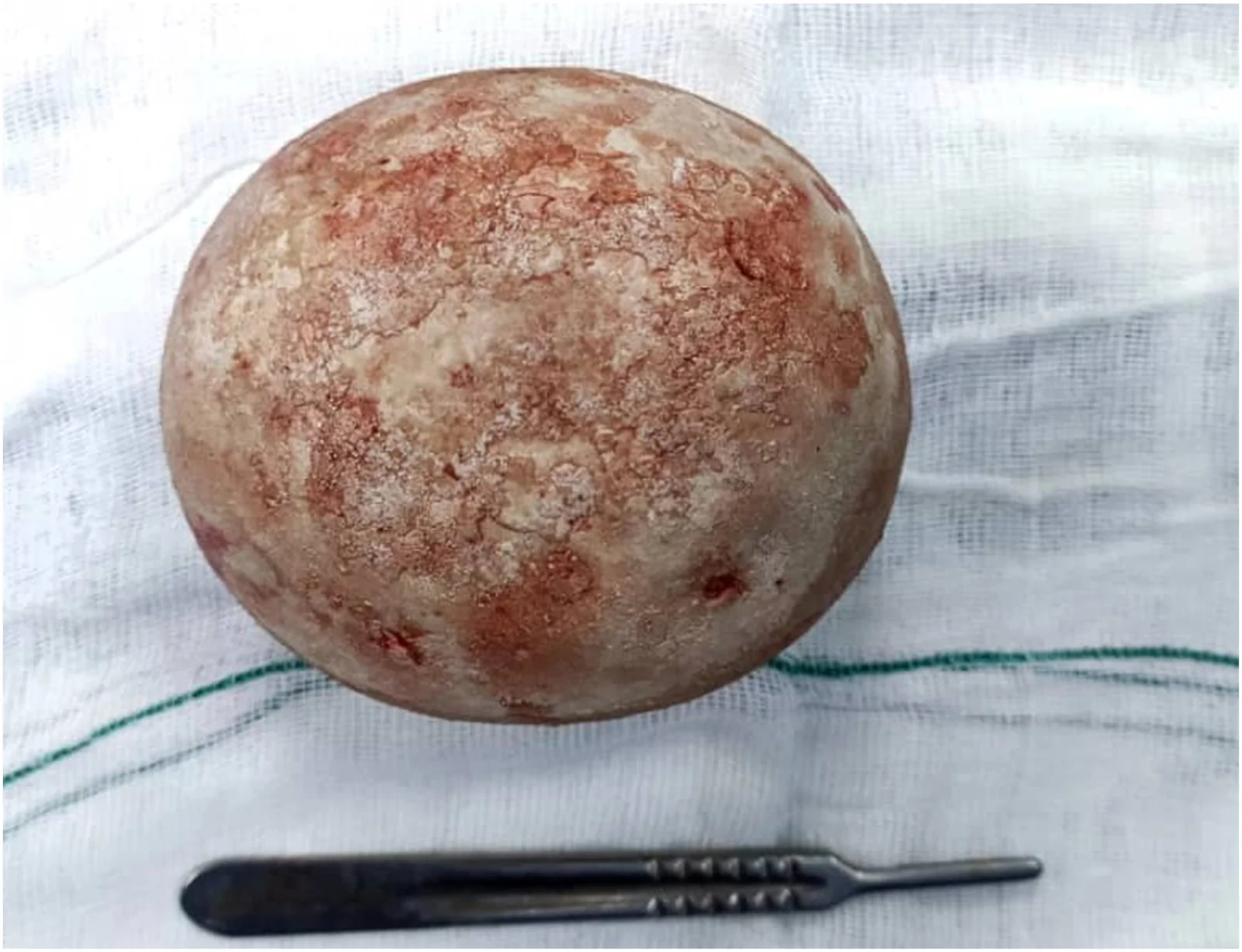
Giant bladder stone weighing 610 g following cystolithotomy.

Due to lethargy, acidosis, and electrolyte disturbances, the patient underwent emergency dialysis. Following improvement in clinical symptoms and laboratory results, the patient underwent bilateral nephrostomy tube placement. After two days, renal function tests returned to normal. The patient was then taken to the operating room, where cystolithotomy was performed. The stone weighed 610 g (Fig. 3). After placement of both a cystostomy tube and a Foley catheter, followed by wound closure, the patient was transferred to the ward. Stone analysis demonstrated magnesium ammonium phosphate composition. On the second postoperative day, after ensuring the absence of obstruction in the ureteral tract via bilateral nephrostogram, both nephrostomies were removed. Following recovery on the fourth postoperative day, the patient was referred to another center for management of intermittent partial bowel obstruction and, if feasible, enterolysis.

## 3 Discussion

This case is notable for the combination of a giant bladder stone, silent presentation, and severe obstructive renal failure secondary to the bladder stone occurring 15 years after augmentation ileocystoplasty.

Bladder stones account for approximately 5% of all urinary stones. Among these, giant stones — defined as those weighing more than 100 g and measuring over 4 cm — are very rare.^5^ Except for primary or endemic bladder stones, which occur in the absence of underlying urological causes, stone formation in the bladder generally requires a predisposing factor.^1^ Obstructive factors resulting in impaired bladder emptying, as well as infections, are important contributors to bladder stone formation.^6^ Enterocystoplasty and neobladder reconstruction techniques have also been associated with urinary stone formation. Metabolic alterations, mucus production, urinary stasis, bacteriuria, and foreign material within augmented bladders contribute to stone formation.^4^^,^^7^ When stones develop within bowel-segment urinary reservoirs, they are usually detected during routine follow-up before becoming complicated. However, rare cases of a single giant neobladder stone have been reported, most often occurring 9-14 years after surgery.^4^^,^^8^ In a notable recent article, Hiruma et al. reported a 5 kg neobladder stone discovered 25 years after radical cystoprostatectomy.^7^ A summary of previously reported giant calculi occurring in bowel-segment urinary reservoirs is presented in Table 1 ^4^^,^^7^, ^8^, ^9^, ^10^, ^11^, ^12^, ^13^, ^14^, ^15^.

**Table 1:** Summary of previously reported cases of giant stones following bowel-segment urinary reconstruction.

Author, Year	Age/Sex	Original Surgery	Time to Stone Detection (Years)	Size (cm)	Weight (g)	Obstructive Renal Failure
Current Case (2026)	68/M	Ileocystoplasty augmentation	15	10 × 9.8	610	Yes
Hiruma et al., 2025	56/M	Radical cystectomy + Neobladder	25	21.6 × 15.5 × 18.5	5000	No
Maachi et al., 2026	78/M	Radical cystectomy + Ileal neobladder	18	10 × 8 × 6 & 9 × 7 × 8	950 (combined)	No
Gu et al., 2023	70/F	Radical cystectomy + Orthotopic ileal neobladder	14	13 × 11.5 × 9	903	Yes
Abrol et al., 2011	32/M	Radical cystectomy + Sigmoid neobladder	7	10 × 8 × 6 & 10 × 7 × 6	815 (combined)	No
Tiu & Soloway, 2014	60/M	Radical cystectomy + Neobladder	Not specified	12	860	No
Nguyen & Choi, 2017	64/M	Radical cystectomy + Orthotopic neobladder	>10	12 × 9.5 × 7.5	770	No
Hatanaka et al., 2008	67/M	Radical cystectomy + Orthotopic neobladder	Not specified	Not specified	108	No
Serni et al., 2004	51/F	Radical cystectomy + Orthotopic ileal neobladder	12	10.5 × 7 × 7	760	No
Lee & Choi, 2026	72/M	Radical cystectomy + Orthotopic neobladder	20	11.0	Not specified	No
Lu et al., 2022	68/M	Radical cystectomy + Orthotopic ileal neobladder	10	9 × 9	Not specified	No

Dysuria, hematuria, and lower urinary tract symptoms are the classic symptoms of bladder stones. Persistent or recurrent urinary tract infections and chronic bladder dysfunction are well-recognized complications of urinary stones.^1^^,^^16^ Bladder stones with associated chronic mucosal injury may also predispose patients to the development of squamous cell carcinoma.^17^ Several studies have additionally examined a significant association between bladder stones and sexual dysfunction.^16^ Obstructive renal failure, however, is a rare complication of bladder stones. Due to the mobility of stones within the bladder cavity, they rarely cause sufficient disruption of urinary flow to result in renal failure. In the article by Molla et al., five case reports from the literature were cited in which giant bladder stones were identified as the cause of renal failure.^1^ The development of renal failure following giant bladder stones after bowel-segment urinary reconstruction is even rarer. Although several cases of giant neobladder stones have been reported (Table 1), the majority presented with lower urinary tract symptoms or abdominal pain. The present case is exceptional in two respects: first, the patient developed severe obstructive renal failure secondary to the giant bladder stone within the reconstructed urinary reservoir, a finding reported in only one prior case^8^; second, and more notably, this life-threatening complication occurred in the complete absence of urinary symptoms. To our knowledge, such presentations appear to be exceedingly rare in the literature. The absence of urinary symptoms in our patient may be attributed to neobladder hypoesthesia, reduced afferent signaling, and the patient's high pain threshold, though such mechanisms remain speculative.

Although regular follow-up after cystoplasty is crucial for preventing subsequent complications, our patient's history of alcohol and opiate addiction, low socioeconomic status, and lack of access to routine healthcare indicate an absence of appropriate postoperative follow-up. Maachi et al. proposed a comprehensive prevention plan based on infection control, adequate hydration, mucosal clearance, and long-term regular follow-up to reduce complications and stone formation following enterocystoplasty and neobladder reconstruction.^4^

## 4 Conclusion

The patient presented in this article highlights the importance of lifelong, regular follow-up after cystoplasty in preventing complications and underscores the possibility of rare but life-threatening complications in the absence of appropriate follow-up. Furthermore, this article discusses renal failure as a rare complication of giant bladder stones. This case emphasizes that giant stones within bowel-segment urinary reservoirs may remain clinically silent until life-threatening obstructive complications occur.

## Ethics

Patient informed consent was obtained to publish his information. The patient's private information remained confidential with the researchers.

## Financial support and sponsorship

None.

## Declaration of competing interest

The authors report no conflicts of interest in this work.
